# Editorial: Rising stars in molecular psychiatry

**DOI:** 10.3389/fpsyt.2023.1125986

**Published:** 2023-01-24

**Authors:** Zhongli Yang, Ming D. Li

**Affiliations:** State Key Laboratory for Diagnosis and Treatment of Infectious Diseases, National Clinical Research Center for Infectious Diseases, National Medical Center for Infectious Diseases, Collaborative Innovation Center for Diagnosis and Treatment of Infectious Diseases, The First Affiliated Hospital, Zhejiang University School of Medicine, Hangzhou, China

**Keywords:** mental disorder, biomarkers, precision medicine, corticotropin-releasing hormone (CRH), smoking

The primary objective of precision medicine in psychiatry is to find biomarkers at different levels which can be used to guide treatment and reduce advance effect of drugs used for the treatment of psychiatric disorders. To reach this ultimate goal, it is essential to promote more and more young generation scientists to do cutting-edge psychiatric-related research. Under this scenario, this Research Topic aims to gather innovative research studies on molecular psychiatry, studies that range from searching potential biomarkers at RNA (Long et al.) and protein (Hylen et al.) levels to understand the regulatory mechanism linking mQTLs and eQTLs to phenotype (Yang et al.) and to the study dedicated to determine how CRH-expressing neurons are associated with MDD (Oh et al.). Additionally, a fifth manuscript has been published in this Research Topic, which describes how to reduce adverse drug reactions by utilizing information from polypharmacy and pharmacogenetics (Mostafa et al.).

The first biomarker study in the current Research Topic was reported by Long et al., which analyzed two datasets from the Gene Expression Omnibus database (GSE53987, GSE98793) for Major depressive disorder (MDD) by using various analytical tools which include weighted gene co-expression network analysis, a Venn diagram, and receiver operating characteristic curve analysis. Through these analyses, the authors found 163 common differentially expressed genes (DEGs) between the two datasets and 17 candidate hub genes for MDD. From these candidate hub genes, the authors then performed logistic regression and receiver operating characteristic curve analysis, which showed that the combination of *CEP350, SMAD5*, and *HSPG2* has a relatively high diagnostic value for MDD. However, although the authors provided reasonable reasons for potential involvement of the three nominated candidate genes in the pathogenesis of MDD according to their analytical results and literature search, the detailed molecular mechanism and clinical applications of them remain to be further investigated in the future by using both *in vivo* and *in vitro* methods.

The second research article featured in the current Research Topic dealt with the understanding how inflammation and metabolism are related to mental disorders. Previous research has established a connection between inflammation and mental disorders and individuals with mental disorders. However, the interplay between inflammation and metabolism in severe mental disorders remains unclear. In this study, after analyzing the lipidomic profile in plasma between 39 patients with schizophrenia, autism, obsessive-compulsive disorder and non-suicidal self-injury disorder and 39 age- and sex-matched healthy controls, Hylén et al. showed that two particular triglycerides and one ether phospholipid were associated with dysregulated inflammation, and these lipid perturbations were specifically linked to the inflammatory markers osteopontin and IL-1 receptor antagonist. This report suggests that individuals with different mental disorders might share some common immunemetabolic pathways, which could be explored as potential therapeutic targets for the treatment of these disorders in future with large studies.

The third study in this Research Topic dealing with the connection from genetic variants, RNA expression, DNA methylation to smoking phenotype was authored by Yang et al. In the past, although various susceptibility genes have been revealed to influence tobacco smoking, the underlying regulatory mechanisms between genetic variants and smoking are poorly understood. To attack this issue, Yang et al. investigated *cis*-expression quantitative trait loci (*cis*-eQTLs) and methylation quantitative trait loci (mQTLs) for 56 candidate smoking-linked genes based on their previous report (1) by using the BrainCloud cohort samples. They found an eQTL to affect *EGLN2* expression in the European sample and two mQTLs to be detected in CpG sites in *NRXN1* and *CYP2A7*. Further, they found that the minor allele of SNP rs3745277 in *CYP2A7P1* (downstream of *CYP2B6*) reduced methylation at the CpG site (i.e., cg25427638) for *CYP2A7* and expression of *CYP2B6*, and had a small proportion in smokers relative to non-smokers (8.8 vs. 42.3%; OR = 0.14, 95% CI: 0.02–0.62; *P* = 4.47 × 10^−3^) in a dominant way. One of the important aspects of this report is that this study revealed a regulatory mechanism linking mQTLs to the smoking phenotype by analyzing genetic variation, DNA methylation, mRNA expression, and smoking status together with the same participants. However, in the consideration of small sample size used in the study, these findings remain to be validated in a large postmortem sample.

The fourth study of this Research Topic dealt with the determination of how CRH-expressing neurons are associated with MDD. Based on the findings from a transcriptome meta-analysis where significantly lower expression of corticotropin-releasing hormone (CRH) mRNA was detected in corticolimbic regions of MDD patients, this suggests that cortical CRH-expressing (CRH+) cells are affected in MDD (2). However, the characteristic features of CRH+ cells in human brain cortex and their association with MDD are largely unknown. By using laser-capture microdissection and RNA-seq techniques to assess potential biological functions affected in CRH+ GABAergic interneurons in a subset of MDD subjects with characterized reduced CRH expression, Oh et al. found that about 80% CRH+ cells were GABAergic and 17.5% were glutamatergic. They further found that MDD subjects displayed lower CRH mRNA levels in GABAergic interneurons relative to comparison subjects without changes in cell density. These findings indicate that CRH+ cells in human sgACC are a heterogeneous population of GABAergic interneurons, although largely co-expressing VIP, suggesting that MDD is associated with reduced markers of inhibitory function in sgACC CRH+ interneurons. In the consideration of a small cohort investigated, this study should be considered as exploratory and hypothesis-generating. Further, it is also interested to know whether same findings could be obtained from female MDD subjects as we know there are sex differences in the prevalence and pathology of MDD.

The last study included in this Research Topic, by Mostafa et al., focused on the precision medicine in psychiatry. Polypharmacy (defined as the use of five or more regular medicines) and genetic variants that strongly influence medication response are two major risk factors for adverse drug reactions. To evaluate the extent of phenoconversion and its potential impact on the reporting of pharmacogenetics results in a cohort of acute aged persons mental health patients on polypharmacy, Mostafa et al. analyzed 137 acute aged persons mental health patients with genetic variants data for CYP2D6, CYP2C19, and CYP2C9 enzymes and medication data at admission and discharge. They found that aged persons mental health patients are commonly prescribed medications with actionable pharmacogenetics guideline recommendations. This study suggests that interpretation of these recommendations must consider the effects of phenoconversion.

## Author contributions

Both authors listed have made a substantial, direct, and intellectual contribution to the work and approved it for publication.
